# Internet-Delivered Interventions for Depression and Anxiety Symptoms in Children and Young People: Systematic Review and Meta-analysis

**DOI:** 10.2196/33551

**Published:** 2022-05-12

**Authors:** Nora Eilert, Rebecca Wogan, Aisling Leen, Derek Richards

**Affiliations:** 1 e-Mental Health Group School of Psychology University of Dublin Trinity College Dublin Ireland; 2 SilverCloud Science SilverCloud Health Dublin Ireland

**Keywords:** internet-delivered interventions, children and young people, depression, anxiety, digital health

## Abstract

**Background:**

Mental health difficulties in children and adolescents are highly prevalent; however, only a minority receive adequate mental health care. Internet-delivered interventions offer a promising opportunity to increase access to mental health treatment. Research has demonstrated their effectiveness as a treatment for depression and anxiety in adults. This work provides an up-to-date examination of the available intervention options and their effectiveness for children and young people (CYP).

**Objective:**

In this systematic review and meta-analysis, we aimed to determine the evidence available for the effectiveness of internet-delivered interventions for treating anxiety and depression in CYP.

**Methods:**

Systematic literature searches were conducted throughout November 2020 using PubMed, PsycINFO, and EBSCO academic search complete electronic databases to find outcome trials of internet-delivered interventions treating symptoms of anxiety and/or depression in CYP by being either directly delivered to the CYP or delivered via their parents. Studies were eligible for meta-analysis if they were randomized controlled trials. Risk of bias and publication biases were evaluated, and Hedges *g* between group effect sizes evaluating intervention effects after treatment were calculated. Meta-analyses used random-effects models as per protocol.

**Results:**

A total of 23 studies met the eligibility criteria for the systematic review, of which 16 were included in the meta-analyses, including 977 participants in internet-delivered treatment conditions and 1008 participants in control conditions across 21 comparisons. Random-effects models detected a significant small effect for anxiety symptoms (across 20 comparisons; Hedges *g*=−0.25, 95% CI −0.38 to −0.12; *P*<.001) and a small but not significant effect for depression (across 13 comparisons; Hedges *g*=−0.27, 95% CI −0.55 to 0.01; *P*=.06) in favor of internet-delivered interventions compared with control groups. Regarding secondary outcomes, there was a small effect of treatment across 9 comparisons for impaired functioning (Hedges *g*=0.52, 95% CI 0.24-0.80; *P*<.001), and 5 comparisons of quality of life showed no effect (Hedges *g*=−0.01, 95% CI −0.23 to 0.21; *P*=.94).

**Conclusions:**

The results show that the potential of internet-delivered interventions for young people with symptoms of anxiety or depression has not been tapped into to date. This review highlights an opportunity for the development of population-specific interventions and their research to expand our current knowledge and build an empirical base for digital interventions for CYP.

**Trial Registration:**

PROSPERO CRD42020220171; https://www.crd.york.ac.uk/prospero/display_record.php?RecordID=220171

## Introduction

### Background

Depression and anxiety disorders rank high among the difficulties for children and young people (CYP), contributing to significant disability, and are associated with lasting intellectual, academic, and social impairment [1-3]. Mental health issues, which often first present at a young age [4], can contribute to lifelong physical health difficulties and reduced quality of life in adulthood [5,6]. Failure to access treatment at such a critical developmental stage can result in serious negative consequences for functioning or even long-term disability [7]. Despite the high prevalence rates and worldwide recognition of the importance of strengthening mental health in CYP, demand still surpasses the capacity of services [8] and only a minority receive adequate mental health care [9]. In one study, after 4 weeks of referral, only 20% received treatment [10], presenting a major issue, as long waiting times have been associated with poorer outcomes, worsening of symptoms, and a greater chance of families disengaging from treatment [11].

Global inadequacies in the provision of mental health services have been attributed to three areas: access to services, implementation, and policy issues [9]. In England, mental health services for CYP are historically underfunded, seeing service cuts of up to 75% even as demand increases at a rate of approximately 11% per annum [12]. One core issue of access, which inhibits the expansion of service provision, is the scarcity of highly trained therapists and supporters to deliver therapeutic content [13], especially in relation to CYP [14]. In a 2017 review of mental health services for CYP in the United Kingdom, CYP reported concerns that the staff members were not adequately trained to meet their needs [15]. Additional personal barriers to young people receiving care can involve location-based or finance-based inaccessibility of services, feelings of embarrassment and perceived stigma, desire to be more self-reliant, and difficulties in recognizing mental health concerns [16].

Internet-delivered interventions are an increasingly popular way to address some of the barriers to access owing to their scalability, efficiency, and potential for personalization [17]. Furthermore, given young people’s familiarity and avid consumption of technology and the internet [18], digital interventions present a possible way to reach larger numbers of young people. Young people use the internet to access information regarding mental health issues, to obtain support for issues when in need, and to connect with peers [19]. In addition to increasing access to care, the use of technology-based treatments has improved patient and family outcomes and quality of life [9]. Evidence has shown comparable effectiveness of internet-delivered interventions and face-to-face brief psychological interventions in treating depression and anxiety in adults (Palacios J, unpublished data, April 2022) [20] and some evidence for their use in CYP [21].

In general, studies of internet-delivered interventions for CYP have lagged behind adult equivalents, and previous systematic reviews have sought to address this [22,23]. However, the update from Grist et al [22] included any technology-delivered treatments, such as video games, which can vary substantially in the mode of delivery and support provided, not to mention design or psychotherapeutic approach, and therefore likely have different effects or mechanisms of change [13]. Hollis et al [17] also conducted a metareview including young adults aged ≥18 years but ≤25 years and defined broad inclusion criteria for therapy delivered over technological devices, such as CD-ROM, SMS text messaging, or videoconferencing. Considering the broad and highly varied nature of the interventions under this rubric, Hollis et al [17] recommended evaluating evidence-based, *core* components of digital-delivered interventions (ie, active ingredients of interventions associated with uptake, adherence, and clinical outcomes).

### Objectives

It is still not clear whether internet-delivered interventions are effective in treating depression and anxiety in CYP. Internet-delivered mental health interventions are rapidly advancing; therefore, we conducted a systematic review to provide an up-to-date analysis of the available intervention options and their effectiveness. Specifically, the aims of this systematic review are (1) to evaluate the current state of evidence for the effectiveness of internet-delivered interventions for childhood and adolescence anxiety and depression symptoms and (2) to assess whether internet-delivered interventions are effective in treating symptoms of anxiety and depression in children and adolescents.

## Methods

### Literature Search

This systematic review and meta-analysis was completed in line with the PRISMA (Preferred Reporting Items for Systematic Reviews and Meta-Analyses) statement [24]; for the corresponding checklist, refer to Multimedia Appendix 1. Its protocol was prospectively registered at PROSPERO (reference number CRD42020220171). A systematic literature search for English language articles was conducted in early November 2020, and the final searches were conducted on November 19, 2020, across three electronic databases: PubMed, PsycINFO, and EBSCO academic search complete. Each database was searched individually with search terms specified by population, presenting the problem, intervention, and intervention medium (refer to Table 1 for examples of the search terms used across databases). In addition, we reviewed references of other relevant review papers, checked trial registers for recent publications related to eligible trial protocols identified through the database searches, and drew on the expertise of the last author (DR) in the field to identify publications that may have been missed.

**Table 1:** Examples of search terms used across databases.

Search category	Examples of search terms
Population	*Child*^a^ or *adolescent*^a^ or *parent*^a^ or *parenting*^a^
Presenting problem	*Depression*^a^ or *anxiety*^a^ or *mental health*^a^
Intervention	*Therapeutics*^a^ or *psychotherapy*^a^ or *intervention*^b^ or *psychoeducat*^b^ or *manag*^b^ or *train*^b^
Medium	*Internet*^a^ or *computer*^b^ or *web*^b^ or *online*^b^ or *technolog*^b^ or *phone application*^b^ or *app*^b^ or *mobile*^b^

^a^MeSH (Medical Subject Headings) term.

^b^Key concept in PsycINFO or title term in PubMed.

### Selection of Studies

After duplicates were deleted via the reference manager Mendeley, entirely off-topic studies were excluded based on the title by one researcher (AL). Eligibility screening of the remaining papers was conducted by two researchers (NE and AL), and discrepancies were resolved via discussion between the researchers and consultation with a senior researcher (DR). The grounds for exclusion of studies were recorded according to a predefined hierarchy.

### Eligibility Criteria

To be included in the systematic review, studies had to (1) be outcome studies, providing at least pretreatment and posttreatment clinical outcome data pertaining to anxiety or depression; (2) be implementing a transdiagnostic or disorder-specific low-intensity intervention delivered remotely via the internet (eg, high-intensity interventions such as videoconference psychotherapy or CD-ROM–based interventions were excluded), targeting symptoms of anxiety and/or depression in children or young people (intervention could be delivered directly to the child or young person or via their parents or guardians); (3) report the average age of the CYP, for whose symptoms the intervention was primarily intended, to be aged ≤18 years; and (4) have only included CYP who presented with symptoms of anxiety and/or depression (individual study inclusion criteria needed to include current symptoms of anxiety and/or depression assessed via self-report measures or clinical interviews). Furthermore, to be included in the meta-analysis, studies needed to be individually randomized controlled trials (RCTs).

### Data Extraction

Extracted data from studies included (1) participant characteristics (percentage female, mean age, and age range of the sample), (2) study characteristics (country of setting, recruitment strategy, clinical eligibility criteria implemented, and type of control group), (3) intervention characteristics (intervention focus of anxiety and/or depression, intervention delivery to youth and/or parents, intervention theoretical orientation, intervention support delivered, number of modules in intervention and length of treatment, and average amount of the intervention completed by participants), and (4) means and SDs or equivalent intention-to-treat metrics facilitating the calculation of posttreatment and follow-up between-group effect sizes where applicable. Data were extracted by one researcher (AL or RW) and checked for accuracy by another researcher (NE).

For outcome data extraction, we created a hierarchy of instruments for our constructs of interest (primary: depression and anxiety; secondary: impaired functioning and quality of life) before data extraction to facilitate uniformity for studies implementing multiple measures for the same construct. Each hierarchy was composed of a list of relevant outcome measures ranked by their properties of interpretability, reliability, and validity. Given the primary interest in generic anxiety in this meta-analysis, generic anxiety measures were given preference over disorder-specific ones (eg, in 1 study [25], the Beck Anxiety Inventory was selected over the Social Phobia Screening Questionnaire for Children). Where there were multiple forms completed of the same measure (eg, Spence Children’s Anxiety Scale-Parent Version and Child Version), the scores were averaged.

### Quality Assessment of Included Studies

The risk of bias was determined for each study included in the meta-analysis based on the CLEAR NPT (Checklist to Evaluate a Report of a Nonpharmacological Trial) checklist [26], which evaluates the quality of RCTs addressing nonpharmacological trials. This checklist has been successfully used in previous meta-analytic studies of internet-delivered interventions for depression [27] and anxiety [28]. This checklist features 10 questions and 5 subquestions, predominantly requiring an answer of yes, no, or unclear. The questions concerned the adequacy of randomization; availability of details of the interventions; appropriateness of supporters’ skills; treatment adherence measurement; blinding of those involved or, if not, notification of steps taken to prevent bias; consistency across conditions’ follow-up schedules; and whether an intention-to-treat principle of analysis was followed. Two researchers (RW and AL) independently completed the CLEAR NPT checklist for all studies on the primary outcomes of depression and anxiety. Conflicts were resolved by checking and discussing the given study and, if further clarity was required, by consulting the first author (NE). Risk of bias assessments were detailed narratively rather than incorporated into meta-analytic models, as the CLEAR NPT checklist does not provide an overall degree of study quality.

### Meta-analytic Procedures

Effect sizes were calculated either from observed means and SDs or estimated marginal means, SEs, and Cohen *d*, if available, with the latter taking precedence if both were reported within a given study. The formulas provided by Borenstein et al [29] were used to calculate Hedges *g* and its SE. In 3-arm trials in which either both active arms met the inclusion criteria or 2 different types of control groups were implemented, the sample sizes of the group that was to be entered into the analysis twice was halved to allow for the calculation of separate effect sizes by trial arm [30]. For effect size, we implemented the following cut-off points: 0-0.32 for a small effect, 0.33-0.55 for a moderate effect, and 0.56-1.2 for a large effect [31].

All analyses were conducted in R (R Foundation for Statistical Computing) using the *meta*, *metafor*, and *dmeta* packages [32-34]. In line with the protocol [35], random-effects models were used to pool effect sizes to account for the anticipated moderate to high levels of between-study heterogeneity. Between-study variance was estimated via restricted maximum likelihood, and heterogeneity was assessed using the *Q* value, *I²* statistic, and prediction intervals (PIs). According to Higgins and Thompson [36], an *I²* value of 0% indicates no heterogeneity, 25% indicates low heterogeneity, 50% indicates moderate heterogeneity, and 75% indicates high heterogeneity. The presence of outliers and model fit was assessed using diagnostic plots and statistics. Owing to the inclusion of studies with narrow and wide focus on intervention aim and content, forest plots detailing primary outcomes by intervention focus (anxiety, low mood and/or depression, or transdiagnostic) were selected to ensure reporting clarity. In a deviation from the registered protocol [35], follow-up between-group effects were assessed through the same models as posttreatment effects, where this was feasible (ie, a sufficient number of studies included relevant data). Where multiple follow-up time points were available for one study, the longest follow-up time point was selected. To explore various potential moderators, mixed-effect models of primary outcomes (anxiety and depression) using the Knapp-Hartung method to reduce the chance of a type 1 error were conducted. Moderators were only explored statistically if there were at least six moderate to large studies with data available for any continuous moderator and four moderate to large studies per subgroup for categorical moderators [37]. Funnel plots and Egger [38] test were used to explore publication bias.

## Results

### Selection and Inclusion of Studies

**Figure 1:**
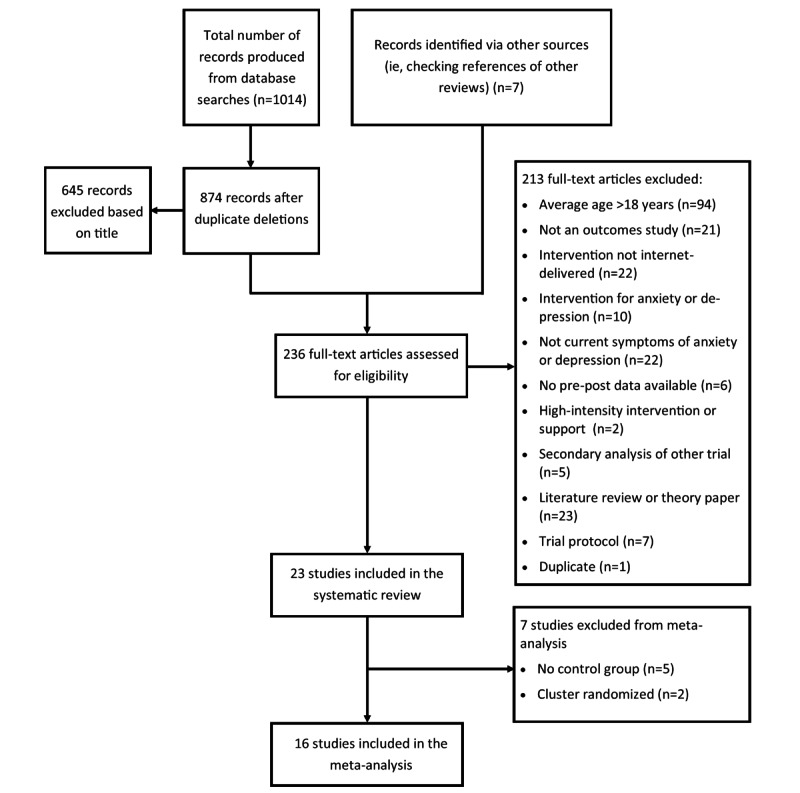
Flow of selection and exclusion of studies.

The database searches resulted in 1014 articles, whereas an additional 11 results were obtained from other sources (refer to the *Literature Search* section). Duplicate removal left 874 articles, 645 of which were excluded based on the title. The full-text versions of the remaining 236 articles were assessed for potential eligibility, of which 213 did not meet the eligibility criteria and were excluded. Finally, 23 studies were deemed eligible for the systematic review, and an additional 7 studies were excluded from the meta-analysis because of not having conducted an RCT. A total of 16 studies met the inclusion criteria for meta-analysis. The study selection process and reasons for exclusion are summarized in Figure 1.

### Description of Included Studies

#### Overview

This review reports 16 RCTs and 7 non-RCTs published in English, the main characteristics of which are presented in Tables 2 and 3. A total of 6981 participants were included, ranging in age from 3 to 21 years and recruited through school, email, flyers, websites, local media (radio, newspaper, etc), social media, youth centers, guardians, or parent groups. A total of 11 studies involved recruitment from different care settings such as general practitioners, mental health professionals, mental health services, and clinics. Moreover, 6 studies were conducted in Sweden; 6 in Australia; 5 in the Netherlands; and 1 each in China, Canada, Denmark, Iran, New Zealand, and the United States.

**Table 2:** Study characteristics, constructs of interest, and outcome measures used across meta-analyses.

Study and country		Sample size (N)^a^	Age, mean (range)	Eligibility criteria	Control (n)	Outcomes included in meta-analysis			
						Anxiety	Depression	Impaired functioning	Quality of life
**Anderson et al, 2012 [39]**									
	Australia	N=132 (female 70, male 62)	12.12 (7-18)	Structured clinical interview	No control group	—^b^	—	—	—
**Conaughton et al, 2017 [40]^c^**									
	Australia	N=42 (female 6, male 36)	9.74 (8-12)	Structured clinical interview	Wait-list (n=21)	SCAS-C/P^d^	—	CGAS^e^-clinician rated	—
**de Voogd et al, 2017 [41]^c^**									
	The Netherlands	N=119 (female 75, male 44)	15.68 (12-18)	>16 SCARED^f^ or >7 CDI^g^	Placebo (n=39)	SCARED	CDI	—	—
**de Voogd et al, 2017 [42]^c^**									
	The Netherlands	N=108 (female 72, male 36)	14.45 (11-19)	>16 SCARED or >7 CDI	Placebo (n=32), waitlist (n=38)	SCARED	CDI	—	—
**Hoek et al, 2012 [43]^c^**									
	The Netherlands	N=45, (female 34, male 11)	16.07 (12-21)	Self-report (mild to moderate depressive and/or anxiety symptoms, NIMH DISC-IV^h^)	Wait-list (n=23)	HADS-A^i^	CES-D^j^	—	—
**Ip et al, 2016 [44]^c^**									
	China	N=257 (female 175; male 82)	14.6 (13-17)	CESD-R score of 12-40	Attention control (n=127)	DASS-21^k^ anxiety subscale	CESD-R	—	—
**Jolstedt et al, 2018 [45]^c^**									
	Sweden	N=131, (female 70, male 61)	9.75 (8-12)	>Moderate anxiety disorder diagnosis	Web-based child-directed play (n=65)	RCADS^l^-child and parent rated	—	CGAS-clinician rated	KIDSCREEN^m^-child and parent rated
**Lindqvist et al, 2020 [46]^c^**									
	Sweden	N=76 (female 61, male 15)	16.6 (15-18)	Unipolar major depressive disorder diagnosis (≥10 on the QIDS-A17-SRs^n^)	Web-based supportive contact (n=38)	GAD-7^o^	QIDS-A17-SR	—	—
**March et al, 2018 [47]**									
	Australia	N=4425 (female 2938, male 1406, other=81)	12.95 (7-17)	≥84th percentile or *t* score ≥60 on the CAS-8^p^	No control group	—	—	—	—
**Moeini et al, 2019 [48]**									
	Iran	N=128 (female 128)	16.2 (15-18)	CES-D score of 10-45	Cluster-randomized control only	—	—	—	—
**Morgan et al, 2017 [49]^c^**									
	Australia	N=433 (female 228, male 205)	4.8 (3-6)	Temperamental inhibition (>30 on the Approach subscale of the STSC^q^)	Wait-list (n=218)	PAS-R^r^-parent rated	—	CALIS-PV^s^-parent rated	—
**Reuland and Teachman, 2014 [50]**									
	United States	N=18 (female 13, male 5)	13 (10-15)	Structured clinical interview	No control group	—	—	—	—
**Rickhi et al, 2015 [51]^c^**									
	Canada	Adolescent subgroup only N=31 (female 26, male 5)	15.3 (12-18)	CDRS-R^t^ score of 40-70	Wait-list (n=13)	—	CDRS-R	—	—
**Silfvernag et al, 2015 [52]**									
	Sweden	N=11 (female 6, male 5)	16.8 (15-19)	Structured clinical interview	No control	—	—	—	—
**Spence et al, 2011 [53]^c^**									
	Australia	N=115 (female 68, male 47)	13.98 (12-18)	Primary diagnosis of generalized anxiety disorder, separation anxiety disorder, social phobia, or specific phobia	Face-to-face CBT^u^ (n=44), wait-list (n=27)	SCAS-C/P	—	CGAS-clinician rated	—
**Spence et al, 2017 [54]^c^**									
	Australia	N=125 (female 75, male 50)	11.29 (8-17)	Structured clinical interview	Wait-list (n=30)	SCAS-C/P	—	CGAS-clinician rated	—
**Sportel et al, 2013 [55]**									
	The Netherlands	N=240 (female 174, male 66)	14.1 (13-15)	Structured clinical interview	Cluster-randomized groups only	—	—	—	—
**Stasiak et al, 2016 [56]**									
	New Zealand	N=42 (female 22, male 20)	11.1 (7-15)	Structured clinical interview	No control group	—	—	—	—
**Stjerneklar et al, 2019 [57]^c^**									
	Denmark	N=70 (female 55, male 15)	15 (13-17)	Structured clinical interview	Wait-list (n=35)	SCAS-C/P	MFQ-S^v^-child and parent rated	CALIS^w^-child rated	WHO-5^x^
**Tillfors et al, 2011 [25]^c^**									
	Sweden	N=19 (female 17, male 2)	16.5 (15-21)	Cutoff for social anxiety disorder (Social Phobia Screening Questionnaire for Children)	Wait-list (n=9)	BAI^y^	MADRS-S^z^	—	QOLI^a^^a^
**Topooco et al, 2019 [58]^c^**									
	Sweden	N=70 (female 67, male 3)	17.5 (15-19)	Depressive symptoms (BDI-II^a^^b^ score ≥14) or major depressive episode as per structured clinical interview	Minimal attention control (n=35)	BAI	BDI-II	—	BBQ^ac^
**Topper et al, 2017 [59]^c^**									
	The Netherlands	N=251 (female 210, male 41)	17.45 (15-22)	Excessive levels of worry and rumination as per population percentile cutoffs	In-person group CBT (n=82), wait-list (n=85)	MASQ-D30^a^^d^ anxiety arousal subscale	BDI-II	—	—
**Vigerland et al, 2016 [21]^c^**									
	Sweden	N=93 (female 51, male 42)	10.1 (8-12)	Structured clinical interview	Wait-list (n=47)	SCAS-C/P	—	CGAS-clinician rated	QOLI-C

^a^Where applicable, sample size is presented as the number male and female participants.

^b^Not available.

^c^Study included in the meta-analysis.

^d^SCAS-C/P: Spence Children’s Anxiety Scale-Parent Version and Child Version.

^e^CGAS: Children’s Global Assessment Scale.

^f^SCARED: Screen for Child Anxiety–Related Emotional Disorders.

^g^CDI: Children’s Depression Inventory.

^h^NIMH DISC-IV: National Institute of Mental Health Diagnostic Interview Schedule for Children Version IV.

^i^HADS-A: Hospital Anxiety and Depression Scale (Anxiety Subscale).

^j^CES-D: Centre for Epidemiologic Studies Depression Scale (also CESD-Revised).

^k^DASS-21: Depression Anxiety and Stress Scale.

^l^RCADS: Revised Children’s Anxiety and Depression Scale.

^m^KIDSCREEN-C/P: Health Related Quality of Life Questionnaire for Children and Young People and their Parents.

^n^QIDS-A17-SR: Quick Inventory of Depressive Symptomatology for Adolescents.

^o^GAD-7: Generalized Anxiety Disorder 7-item scale.

^p^CAS-8: Spence Children’s Anxiety Scale-8 item Version.

^q^STSC: Short Temperament Scale for Children.

^r^PAS-R: Revised Preschool Anxiety Scale.

^s^CALIS-PV: Children’s Anxiety Life Interference Scale-Preschool Version.

^t^CDRS-R: Children’s Depression Rating Scale-revised.

^u^CBT: cognitive behavioral therapy.

^v^MFQ-S: Mood and Feelings Questionnaire.

^w^CALIS: Children’s Anxiety Life Interference Scale.

^x^WHO-5: World Health Organization-Five Well-Being Index.

^y^BAI: Beck Anxiety Inventory.

^z^MADRS-S: Montgomery Åsberg Depression Rating Scale–Self-rated.

^aa^QOLI: Quality of Life Inventory (also QOLI-Child Version).

^ab^BDI-II: Beck Depression Inventory.

^ac^BBQ: Brunnsviken Brief Quality of Life Scale.

^ad^MASQ-D30: Mood and Anxiety Symptom Questionnaire.

**Table 3:** Description of recruitment strategies and interventions used within studies included in meta-analyses.

Study and intervention (n)		Recruitment source	Number of modules and intervention duration	Support	Intervention engagement
**Anderson et al, 2012 [39]**					
	iCBT^a^ for anxiety disorders delivered to youth and parents (n=132)	Recruited via advertising in the media and referrals from guidance officers and mental health professionals	15-16 (10 youth and 5-6 parent modules) over 12 weeks	Weekly emails and one 15-minute telephone call with clinician	Youth completed an average of 8.86 out of 10 sessions; parents completed an average of 4.76 out of 5 or 5.74 out of 6 sessions if assigned 6.
**Conaughton et al, 2017 [40]^b^**					
	iCBT for anxiety disorders delivered to youth and parents (n=21)	Recruited via advertising in the media and referrals from guidance officers, teachers, parents, GPs^c^, and mental health professionals and self-referral	16 (10 youth and 6 parent modules) over 10 weeks	Weekly web-based contact and one short phone call with a therapist	Youth completed an average of 6.7 out of 10 sessions; parents completed an average of 4.86 out of 6 sessions.
**de Voogd et al, 2017 [41]^b^**					
	iCBM^d^ for anxiety and depression delivered to youth (scenario training n=36; picture-based training n=44)	Recruited from 4 secondary schools	8 modules over 4 weeks	No support	Those in scenario training completed an average of 5.56 out of 8 modules, and those in picture-based training completed an average of 5.91 out of 8 reviews.
**de Voogd et al, 2017 [42]^b^**					
	iABM^e^ for anxiety and depression delivered to youth (n=38)	Recruited from 4 secondary schools	8 modules over 4 weeks	No support	Participants completed an average of 5.74 out of 8 modules.
**Hoek et al, 2012 [43]^b^**					
	Internet-based problem-solving therapy for depression and anxiety delivered to youth (n=22)	Recruitment via advertising in schools, mental health clinics, and media and referrals from school doctors	5 modules over 5 weeks	Weekly automated emails and exercise feedback via email by mental health professional and authors	6 participants completed 5 out of 5 modules, 10 completed ≥3, 5 completed 1-2, and 1 participant completed none of the modules.
**Ip et al, 2016 [44]^b^**					
	Integrative iCBT for major depression prevention (CATCH-IT^f^) delivered to youth (n=130)	Recruited from 3 secondary schools (1 all-girls and 2 coeducational schools)	10 modules over 8 months	No support	26 participants completed 10 out of 10 modules; 55 participants completed 5 or more modules.
**Jolstedt et al, 2018 [45]^b^**					
	Exposure-based iCBT for anxiety delivered to parents and youth (n=66)	Recruited via advertising and referrals from mental health services	12 modules over 12 weeks	Weekly asynchronous web-based therapist support	Those in treatment completed an average of 8.91 out of 12 modules.
**Lindqvist et al, 2020 [46]^b^**					
	Internet-based, affect-focused psychodynamic therapy for depression delivered to youth (n=38)	Recruited via advertising on social media, youth centers, and clinics	8 modules over 8 weeks	Web-based feedback and 30-minutes weekly chat with a therapist	Participants completed an average of 5.8 out of 8 modules and attended 6.6 out of 8 chat sessions.
**March et al, 2018 [47]**					
	iCBT for anxiety delivered to youth (n=4425)	Recruited via self-referral, health or education staff, and advertising health information web sites	10 modules over 20 weeks	No support	Average number of modules completed was 2.21 out of 10; 21.65% of participants did not complete the first module.
**Moeini et al, 2019 [48]**					
	iCBT based on social cognitive theory applications for depression delivered to youth (n=64)	Recruited from all-girls high schools	8 modules over 12 weeks	Web-based messages from psychiatrist	—^g^
**Morgan et al, 2017 [49]^b^**					
	iCBT (Cool Little Kids online) for anxiety delivered to parents (n=215)	Recruited via web-based advertising and flyers distributed to preschool services	8 modules over 24 weeks	Support-on-demand (psychologist)	Average number of modules accessed was 4 out of 8.
**Reuland and Teachman, 2014 [50]**					
	iCBM for social anxiety delivered to only youth, only parent, or youth and parents concurrently (n=18)	Recruited via flyers, advertisements, and social networking	8 modules (duration: N/A)	Unsupported with 90-minute group meetings to obtain youth feedback on intervention	—
**Rickhi et al, 2015 [51]^b^**					
	Spirituality-informed e-mental health tool for major depression delivered to youth (n=18)	Adolescent subgroup recruited via email, posters, media, schools, health professionals, and youth organizations	8 modules over 8 weeks	No support	4 out of 31 participants completed less than half of the modules, 2 completed more than half, and 25 completed all modules.
**Silfvernag et al, 2015 [52]**					
	Tailored iCBT for anxiety disorders delivered to youth (n=11)	Referral via guardian, clinic, and self-referral	6-9 modules over 6-18 weeks	Telephone or face-to-face support (if needed)	Average number of modules completed was 5.
**Spence et al, 2011 [53]^b^**					
	iCBT for anxiety disorders delivered to youth and parents (n=44)	Recruited via media advertising and referrals from school guidance officers, GPs, and mental health professionals	15 (10 youth and 5 parent session) over 12 weeks	Email feedback after each session and one 15-minute phone review call by therapist	Average number of sessions completed was 7.5 out of 10 for youth and 4.48 out of 5 for parents.
**Spence et al, 2017 [54]^b^**					
	Social anxiety–specific iCBT (n=47) and generic iCBT for anxiety (n=48) delivered to youth and parents	Recruited via schools, parent groups, mental health professionals, guidance officers, the media, and self-referral	15-16 (10 youth and 5-6 parent sessions) over 12 weeks	Email feedback after each session and one 15-minute phone review call by therapist	Youth completed on average 4-4.75 out of 10 sessions, and parents completed on average 4.32 out of 6 or 3.18 out of 5 sessions.
**Sportel et al, 2013 [55]**					
	iCBM for social anxiety delivered to youth (n=86)	Recruited via 24 schools	20 over 10 weeks	No support	iCBM participants completed on average 8.5 out of 20 sessions.
**Stasiak et al, 2016 [56]**					
	iCBT for anxiety disorders delivered to youth (n=42)	Recruited through referrals from GPs and school public health nurses	15-16 modules (10 youth and 5-6 parent) over 12 weeks	Feedback to child and parent and one 30-minute phone call with therapist	Average number of sessions completed by youth was 4.48 out of 10; it was 4.3 out of 6 for parents of children, and 2.3 out of 5 for parents of adolescents.
**Stjerneklar et al, 2019 [57]^b^**					
	iCBT (ChilledOut online) for anxiety delivered to youth (n=35)	Recruited via advertising and referrals from local health services	8 modules over 14 weeks	Weekly phone calls (average 20 mins) with therapist	Participants completed on average 5.4 out of 8 modules (excluding 2 participants who dropped out).
**Tillfors et al, 2011 [25]^b^**					
	iCBT for social anxiety delivered to youth (n=10)	Recruited via advertising newspapers and in schools	9 modules over 9 weeks	Email feedback after each homework assignment by therapist	Participants finished on average 2.9 out of 9 modules.
**Topooco et al, 2019 [58]^b^**					
	iCBT for depression delivered to youth (n=35)	Recruited via social media posts, schools, youth centers, and clinics	8 modules over 8 weeks	Weekly synchronous therapist support sessions via platform chat feature	Participants completed on average 6.2 out of 8 modules and 5.7 out of 8 chat sessions.
**Topper et al, 2017 [59]^b^**					
	Rumination-focused iCBT for anxiety disorder and major depression prevention delivered to youth (n=84)	Recruited through 13 secondary schools and 2 universities	6 modules over 6 weeks	Clinical psychologist offered feedback after each session	Those who started iCBT completed an average of 3.96 out of 6 sessions; 9.9% did not start iCBT.
**Vigerland et al, 2016 [21]^b^**					
	iCBT for anxiety disorders delivered to both youth and parents (n=46)	Recruited via media advertisement and self-referral	11 (4 youth and 7 parent) modules over 10 weeks	Web-based messages or feedback, 3 phone calls, and optional additional calls with therapist	Average number of modules completed was 9.7 out of 11.

^a^iCBT: internet-delivered cognitive behavioral therapy.

^b^Study included in meta-analysis.

^c^GP: general practitioner.

^d^iCBM: internet-delivered cognitive bias modification.

^e^iABM: internet-delivered attentional bias modification.

^f^CATCH-IT: Competent Adulthood Transition with Cognitive Behavioral Humanistic and Interpersonal Training (Chinese adaptation).

^g^Information not available.

#### Description of Studies Included in the Meta-analysis

Across RCT studies, sample sizes ranged from 19 [25] to 433 [49], with 977 in internet-delivered treatment conditions and 1008 in control conditions. There were 21 comparisons conducted across the 16 RCTs, 13 of which featured a wait-list control and 8 implemented active controls (2 placebo bias modification programs, 2 attention controls, 1 internet-delivered child-directed play, 1 internet supportive contact, 1 face-to-face CBT, and 1 group CBT). A total of 12 active treatment comparisons implemented a form of internet-delivered cognitive behavioral therapy (iCBT), 3 featured internet-delivered cognitive or attentional bias modification interventions (internet-delivered cognitive bias modification [iCBM] or internet-delivered attentional bias modification), 1 implemented a problem-solving therapy, 1 implemented an affect-focused psychodynamic therapy, and 1 implemented a spirituality-informed intervention. Most of these interventions were delivered to CYP, and 5 interventions were also delivered to the parents; however, 1 intervention was delivered to parents only [49]. The RCTs included participants with at least mild to moderate symptoms or those who met the diagnostic criteria for a primary disorder of anxiety or depression assessed via structured clinical interviews or self-report measures. Most studies (12/16, 75%) provided some form of regular scheduled feedback or assistance from a therapist, psychologist, or mental health professional. One study provided support only when requested by participants [49]. Support provided across the range of studies consisted of email, treatment platform chat, or phone calls, or no support. The duration of treatment ranged from 4 weeks [41,42] to 8 months [44], whereas the number of intervention modules ranged from 5, offered to CYP only [43], to 16, offered to both parents and CYP [40,54]. Tables 2 and 3 provide an overview of these findings.

#### Description of Studies Excluded From the Meta-analysis

Of the studies not eligible for inclusion in the meta-analysis (n=7), the sample sizes ranged from 11 [52] to 4425 [47]. A total of 5 studies implemented a form of iCBT and 2 studies implemented iCBM, with treatment periods ranging from 6 [52] to 20 weeks [47], whereas the number of modules ranged from 6 [52] to 20 [55]. All non-RCTs used a clinical measure of the constructs of interest, either in a self-report format or through clinical interviews, to establish their eligibility criteria, which ranged from mild to moderate symptoms of depression or anxiety to diagnosis of clinical symptoms. Most interventions were website-based platforms, and 4 interventions provided some form of support from a qualified or soon-to-be qualified therapist, psychiatrist, or clinician. The aim of support was mainly to offer feedback, motivation, or assistance with the treatment content and consisted of email, webpage messaging, and telephone calls, and 1 study provided face-to-face support if needed. However, 3 non-RCTs were unsupported. The majority of interventions were delivered to CYP, 2 studies delivered the intervention to parents as well as CYP, and 1 of these included a comparison condition delivering the intervention to parents only [50].

### Meta-analysis of Primary and Secondary Outcomes

#### Random-Effects Model for Anxiety

**Figure 2:**
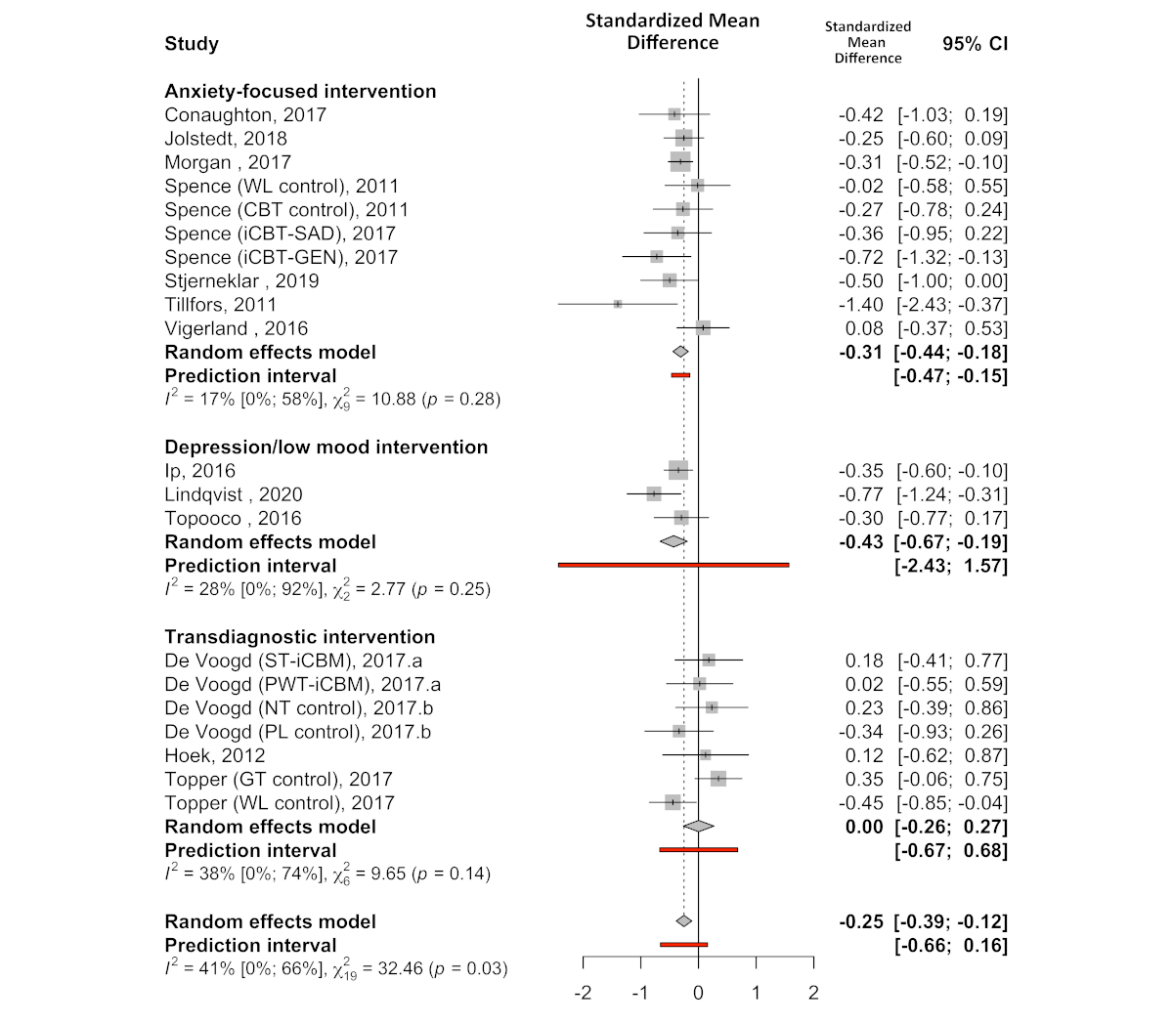
Posttreatment standardized mean difference (Hedges g) between internet-delivered treatment and control groups for anxiety outcomes by intervention focus [21,25,40-46,49,53,54,57-59]. CBT: cognitive behavioral therapy; GT: group treatment; iCBT-GEN: generic internet-delivered cognitive behavioral therapy; iCBT-SAD: internet-delivered cognitive behavioral therapy for social anxiety disorder; NT: no treatment; PL: placebo; PWT-iCBM: picture-word training internet-delivered cognitive bias modification; ST-iCBM: scenario training internet-delivered cognitive bias modification; WL: wait-list.

On the basis of 20 comparisons (across 15 studies), including anxiety-focused interventions (n=8), depression-focused interventions (n=3), and transdiagnostic interventions (n=4), a small effect on anxiety symptoms in favor of internet-delivered treatment was detected (Hedges *g*=−0.25, 95% CI −0.38 to −0.12; *P*<.001). Heterogeneity in the observed effect sizes appeared moderate (Q_19_=32.42; *P*=.03; *I^2^*=41.4%), with the PI crossing the zero line of no effect (95% PI −0.66 to 0.15). Model diagnostics suggested one potential outlier [59] (CBT group-treatment control arm). Excluding this study from the analysis resulted in a reduction of heterogeneity, a moderate effect estimate (Hedges *g*=−0.50, 95% CI −0.40 to −0.20; *P*<.001; Q_18_=23.29; *P*=.18; *I^2^*=22.7%), and a narrower PI no longer crossing zero (95% PI −0.41 to −0.19). Testing for subgroup differences between anxiety-focused interventions, depression-focused interventions, and transdiagnostic interventions revealed significantly different effect estimates by intervention focus (Q_2_=6.13; *P*=.046). Figure 2 [21,25,40-46,49,53,54,57-59] shows the meta-analysis outcomes overall and by intervention focus.

#### Random-Effects Model for Depression

**Figure 3:**
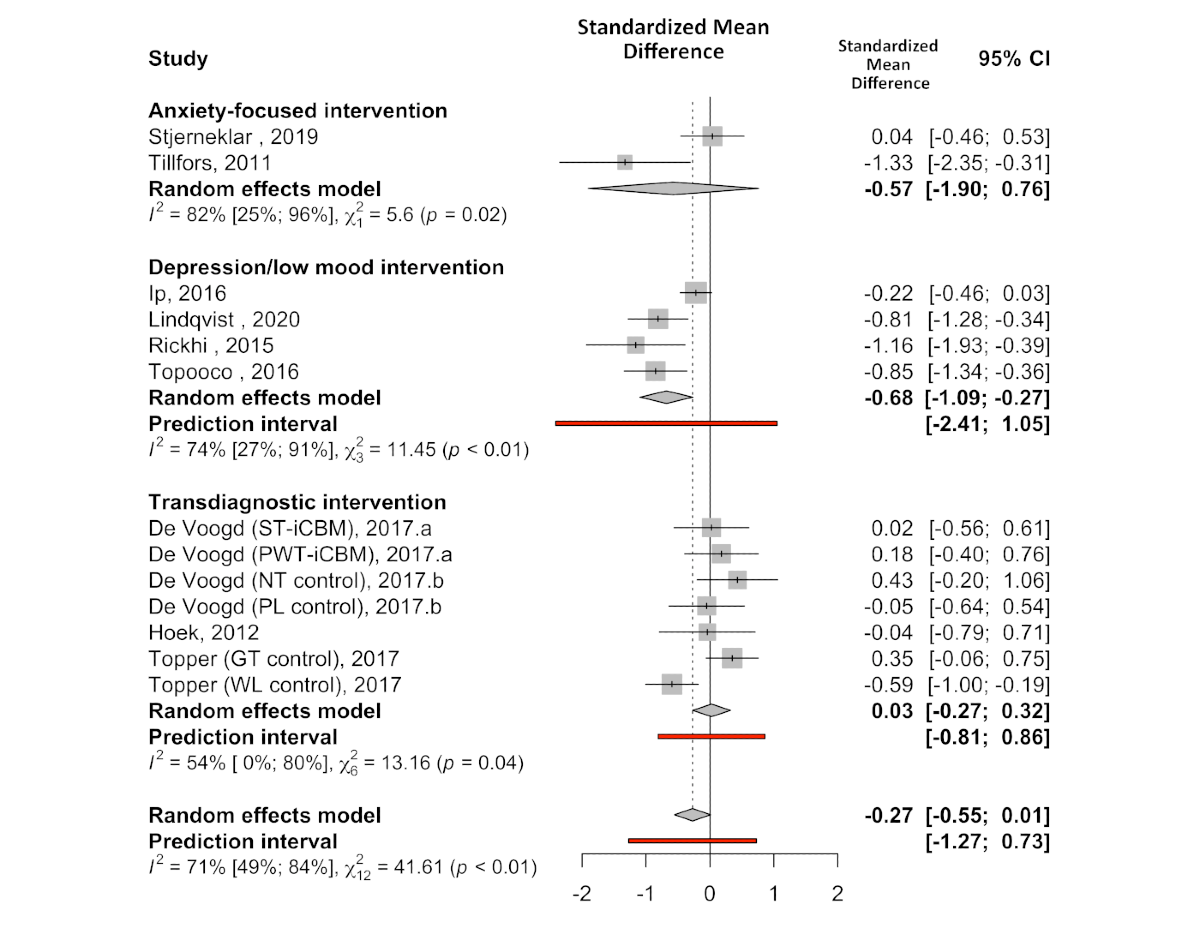
Posttreatment standardized mean difference (Hedges g) between internet-delivered treatment and control groups for depression outcomes by intervention focus [25,41-44,46,51,57-59]. GT: group treatment; NT: no treatment; PL: placebo; PWT-iCBM: picture-word training internet-delivered cognitive bias modification; ST-iCBM: scenario training internet-delivered cognitive bias modification; WL: wait-list.

Drawing on 13 comparisons (across 10 studies) and assessing depressive symptoms in the context of depression or low mood interventions (n=4), anxiety-focused interventions (n=2), and transdiagnostic interventions (n=4), a small effect bordering significance and favoring internet-delivered treatment was observed (Hedges *g*=−0.27, 95% CI −0.55 to 0.01; *P*=.06). There was a high amount of heterogeneity (Q_12_=42.02; *P*<.001; *I^2^*=71.4%), resulting in a wide PI spanning the zero line of no effect (95% PI −1.27 to 0.73). The model diagnostics suggested the absence of outliers. Subgroup analyses suggested that effect sizes differed by intervention focus (Q_2_=7.75; *P*=.02), with depression-focused interventions presenting with the largest effect estimate (Hedges *g*=−0.68, 95% CI −1.10 to −0.27; *P*=.001). Refer to Figure 3 [25,41-44,46,51,57-59] for further details.

#### Random-Effects Model for Impaired Functioning

The overall effect of treatment on levels of functioning across the 9 comparisons (7 studies) was moderate (Hedges *g*=0.52, 95% CI 0.24-0.80; *P*<.001). Heterogeneity was moderate to high (Q_8_=23.27; *P*=.003; *I^2^*=65.6%), and the PI was wide (95% PI −0.38 to 1.43). One potential outlier [40] was detected, whose removal resulted in a smaller effect estimate (Hedges *g*=0.38, 95% CI 0.22-0.54; *P*<.001) but significantly improved heterogeneity (Q_7_=9.25; *P*=.16; *I^2^*=29.6%, 95% PI 0.06-0.70). Refer to Figure S1 in Multimedia Appendix 2 [21,40-45,53-55,57,59] for further details.

#### Random-Effects Model for Quality of Life

In terms of quality of life, outcomes across the 5 comparisons (5 studies) detected no significant effect of treatment (Hedges *g*=−0.01, 95% CI −0.23 to 0.21; *P*=.94). The *I^2^* and *Q* value metrics suggested little heterogeneity (Q_4_=4.70; *P*=.32; *I^2^*=14.9%, 95% PI −0.41 to 0.39), and model diagnostics suggested no outliers. Refer to Figure S2 in Multimedia Appendix 2 for further details.

#### Meta-analysis of Follow-up Outcomes

For anxiety outcomes, pooling of 7 follow-up effect sizes (across 4 studies) revealed no significant effect (Hedges *g*=−0.17, 95% CI −0.58 to 0.24; *P*=.42), with no heterogeneity or outliers detected (Q_6_=0.70; *P*=.99; *I^2^*=0%, 95% PI −0.71 to 0.37). Refer to Figure S3 in Multimedia Appendix 2 for further details. A similar picture emerged for depression outcomes across 8 comparisons (5 studies). The effect estimate remained insignificant (Hedges *g*=−0.18, 95% CI −0.39 to 0.03; *P*=.09), heterogeneity was low (Q_7_=7.02; *P*=.43; *I^2^*=0.4%, 95% PI −0.61 to 0.25), and no outliers were detected. Refer to Figure S4 in Multimedia Appendix 2 for further details.

### Moderator Analyses

Moderator analyses suggested a relationship between the percentage of a study’s sample that was identified as female and the depression effect sizes observed in these studies (*F*_1,11_=6.04; *P*=.03). Higher percentages of females in the study were associated with larger between-group effect sizes for depression outcomes (*b*=−0.03, SE=0.01), accounting for 33.93% of the observed heterogeneity. All other moderator analyses were either insignificant (refer to Table S1 in Multimedia Appendix 2) or not feasible owing to a limited number of studies falling into specific categories (ie, intervention delivered to parents only, iCBM intervention, and face-to-face treatment control group).

### Quality of Studies

#### Risk of Bias

Following screening based on the criteria outlined in the CLEAR NPT checklist, the methodological quality assessment ratings were satisfactory across the included studies. The allocation sequence generation was considered adequate in 88% (15/17) of the studies; all studies provided clear descriptions of the intervention administered, and all studies quantitatively assessed participant adherence. All but one study [21] analyzed the outcomes using an intention‐to‐treat principle. The percentage of studies that adequately detailed their allocation concealment method was 82% (14/17). Clear documentation that care providers had appropriate experience or skill was given in 82% (14/17) of the studies, whereas for 18% (3/17) of the studies, this remained unclear.

As, by their nature, nonpharmacological trials and self‐report outcome measures do not facilitate adequate blinding of participants, care providers, or outcome assessors to treatment allocation, this was often not feasible within the included studies. Therefore, the associated checklist items had the lowest quality ratings. Participants and care providers were blinded in only 18% (3/17) of the studies, with attempts made to blind outcome assessors in 65% (11/17) of studies. To aid in minimizing the risk of bias associated with inadequate blinding, subitems on the CLEAR NPT assessed the following items: for studies in which participants and care providers were not blinded, the provision of all other treatments and care in each randomized group were the same within 79% (11/14) of studies, and the number of participants withdrawn or lost to follow-up was the same in 71% (10/14) of studies. Where outcome assessors were not blinded, none of the studies provided a clear description of the specific methods used to avoid ascertainment bias, that is, systematic differences in outcome assessment. Only 53% (9/17) of studies adhered to the same follow-up schedule for randomized groups, with discrepancies often related to the provision of treatment to wait-list groups, perhaps owing to ethical considerations regarding the withholding of treatment. Refer to Figure S5 in Multimedia Appendix 2 for the quality assessment ratings of the included studies.

#### Publication Bias

Neither funnel plots nor Egger tests suggested the presence of any significant publication bias for anxiety (Egger funnel plot asymmetry: t_18_=−0.29; *P*=.77) and depression outcomes (t_11_=−0.96; *P*=.36). Refer to Figures S6A and S6B in Multimedia Appendix 2 for further details. Owing to fewer studies addressing functional impairment and quality of life, it was not feasible to assess publication bias across these constructs.

## Discussion

### Principal Findings

The study sought to evaluate the state of published evidence for the effectiveness of internet-delivered interventions in treating symptoms of anxiety and depression in CYP compared with control groups. We identified 23 studies of adequate quality examining internet-delivered treatments for anxiety and/or depression in CYP; only 16 of these were RCTs, and hence, they were included in the meta-analysis. Across these controlled comparisons, the anxiety posttreatment effect sizes were small (Hedges *g*=0.3) and favored internet-delivered interventions. Depression outcomes were mixed, with the overall effect estimate based on anxiety-focused interventions, depression-focused interventions, and transdiagnostic interventions remaining insignificant. Among low mood and depression-specific interventions, the effect estimate was significant and large (Hedges *g*=0.7), but given the limited number of studies (n=4) falling into this subgroup, this finding should be considered preliminary. With regard to secondary outcomes, internet-delivered interventions were associated with moderate benefits (Hedges *g*=0.5) in overall levels of functioning; however, no such effects were observed in terms of quality-of-life outcomes. Among the few studies that included controlled follow-up comparisons, there was no evidence for the continuance of the effects of internet-delivered interventions on anxiety and depression symptoms into follow-up.

### Findings in the Context of Previous Reviews

The small to moderate effect sizes observed in this study are somewhat smaller than those reported by previous meta-analyses comparing technology-delivered interventions with wait-list controls (Hedges *g*=0.45-0.68 [21,22]) but more in line with equivalent comparisons against active control groups (Hedges *g*=0.07-0.29 [21,22]). Overall, this study appears to paint a more pessimistic picture of the potential of internet-delivered interventions as they currently stand than previous meta-analyses. Specifically, the nonsignificant improvements in depression symptoms are in contrast with the previous findings of depression treatment effects (Hedges *g*=0.56 [23]). Heterogeneity was considerable within the posttreatment analyses, especially regarding depression outcomes, which may have been due to the wide range of interventions included in the analyses on the one hand and the limited number of studies including depression-focused interventions on the other hand. Indeed, only the posttreatment anxiety outcome was assessed over a comprehensive number of studies and thus might be considered relatively robust (15 studies: 20 comparisons); however, the effect size was so small that one could question the clinical utility of the interventions. With regard to this, we note our more focused inclusion criteria compared with previous meta-analyses, in that we only included internet-delivered interventions (rather than interventions delivered via any form of technology) and we only included studies whose samples presented with current depression or anxiety symptoms (rather than studies that provided population-level or preventive intervention to entire classes in schools, for example). Our preliminary, yet inconclusive, evidence is reminiscent of the review by Hollis et al [17], who noted several methodological limitations preventing them from making definitive conclusions regarding digital health interventions for CYP.

The results of this study do not encourage the effectiveness of digitally delivered interventions for treating symptoms of depression and anxiety in CYP, with robust and consistent between-group effect sizes being a common requirement for the endorsement of digital health interventions in routine care [60]. Furthermore, with little or no evidence for improving the quality of life or having sustained benefits, their utility is questionable. Considering the recent advances in building an evidence base for internet-delivered interventions for anxiety and depression in adults [20,61], this study highlights the lack of equivalent interventions for CYP. Recent systematic reviews and meta-analyses of digitally delivered CBT interventions for depression and anxiety in adults have included over 40 robust trials, yielding strong posttreatment and follow-up effects [27,62]. In contrast, the results from this review and analysis were weak in terms of the number of studies included, their robustness, and the effect sizes we observed. This is surprising given the advances in technology and research [63] as well as the high rates of CYP engagement with technology [64].

Owing to the large heterogeneity in the format of internet-delivered interventions, Vigerland et al [21] outline the apparent uncertainty in the literature regarding the optimal way to treat CYP and recommend consistency in reporting of advantageous factors. Much of the literature supporting internet-delivered interventions focuses on or provides evidence for CBT-based options [21-23], with the evidence sparser for other theoretical perspectives. There is great scope for future research to explore what works for whom in digitally delivered psychological interventions for CYP populations. Further comparisons of specific types of interventions or particular aims of the intervention may be informative and warranted once more research involving this population has been conducted. Here, exploring the differential effectiveness of parent versus CYP-delivered interventions, treatment versus preventive interventions, or between interventions with varying theoretical bases may be particularly interesting. For example, Pennant et al [65] found medium between-group effects for CBT-based digital treatment of symptoms and small effect sizes for prevention across general populations. In contrast, attentional bias modification–based or cognitive bias modification–based interventions were associated with smaller or no effects [22,65].

As mentioned above, increasing access to effective treatment is imperative. It is essential that studies consider accessibility, engagement, and cost-effectiveness. In line with best practice, and as supported iCBT has been associated with larger effects in adults than unsupported iCBT [66], the fact that most included studies provided a form of support is encouraging. However, no studies mentioned any specialist training in delivering treatment to CYP, a potential inadequacy of care.

Furthermore, access of caregivers to the treatment content may have a significant effect on outcomes [22,67], particularly for internet-delivered interventions that involve less therapist support and are usually completed at home, not at a clinic or dedicated practice. Active parental participation was incorporated into 9 of the included studies, whereas, in general, *behind the scenes* parental involvement could encompass any form of social support such as technical help and time management. Parental support given to the CYP completing the intervention may aid engagement with the programs, understanding and learning of content, and the application of new skills [68]. Although this was not a focus of our review, with less than half of the studies directly addressing active parental involvement through delivery to parents alone or in conjunction with the CYP, future reviews should consider the important influence of active and indirect parental involvement on CYP outcomes following internet interventions.

### Limitations

An important limitation of this review was the small number of studies, particularly those assessing depression outcomes. Thus, these results should be interpreted cautiously and may not be a meaningful representation of the potential efficacy of internet-delivered interventions in treating childhood depression and anxiety. Similarly, owing to the limited number of studies, we could not properly evaluate the influence of various moderators, such as the effects of the type of intervention or control group, on our findings. In addition, the effect sizes after treatment were based on only 16 controlled studies, and we did not report any within-group comparisons. Therefore, this evidence must be considered as preliminary. In summary, more high-quality studies analyzing the outcomes for CYP are required. Future studies should include well-established outcome assessments, ensure adequate blinding of participants and outcome assessors when feasible, and balance differences between the treatment and control groups such as posttreatment assessment schedules. Perhaps, this would facilitate more detailed analyses and better estimate specific intervention effectiveness and factors associated with improved outcome analyses, such as type or format of intervention and degree of parental involvement.

### Conclusions

Internet-delivered interventions have the potential to increase the availability and access of CYP to much-needed mental health support. These interventions may be effective, but adult-associated effect sizes are often moderate to large and comparable with face-to-face treatments. This study potentially highlights an insufficient customization of the intervention for CYP needs. This remains a largely underresearched area, and it is important to investigate how interventions can effectively reach and support CYP experiencing anxiety and depressive symptoms. Identifying variables that benefit or interfere with successful treatment outcomes will aid in adapting and enhancing internet-delivered interventions. Future work on the development and research of digital interventions for this population should consider the value of incorporating caregivers and other allied health professionals in the lives of CYP.

## Appendix

Multimedia Appendix 1 PRISMA (Preferred Reporting Items for Systematic Reviews and Meta-Analyses) checklist.

Multimedia Appendix 2 Additional results tables and figures for secondary outcomes (impaired functioning, quality of life, follow-up anxiety and depression outcomes), moderator analysis and study bias assessments.

## Abbreviations

CBT: cognitive behavioral therapy
CLEAR NPT: Checklist to Evaluate a Report of a Nonpharmacological Trial
CYP: children and young people
iCBM: internet-delivered cognitive bias modification
iCBT: internet-delivered cognitive behavioral therapy
PI: prediction interval
PRISMA: Preferred Reporting Items for Systematic Reviews and Meta-Analyses
RCT: randomized controlled trial

## Data Statement

The data set and code used for analysis in this study are available upon request by emailing the corresponding author (NE).
