# Supplementary material for: Internet-Delivered Interventions for Depression and Anxiety Symptoms in Children and Young People: Systematic Review and Meta-analysis
Source: JMIR Pediatr Parent. 2022 May 12;5(2):e33551. doi: 10.2196/33551 (PMC9136650; doi:10.2196/33551)
Supplement: Multimedia Appendix 2 [file pediatrics_v5i2e33551_app2.docx]

# Appendix 2 – Additional tables and figures

## Random-Effect Models

Figure S1. Post-treatment standardized mean difference (Hedge’s g) between internet-delivered treatment and control groups for functioning outcomes. CBT: cognitive behavioral therapy; iCBT-GEN: generic internet-delivered cognitive behavioral therapy; iCBT-SAD: internet-delivered cognitive behavioral therapy for social anxiety disorder; SMD: standardized mean difference; WL: wait-list.


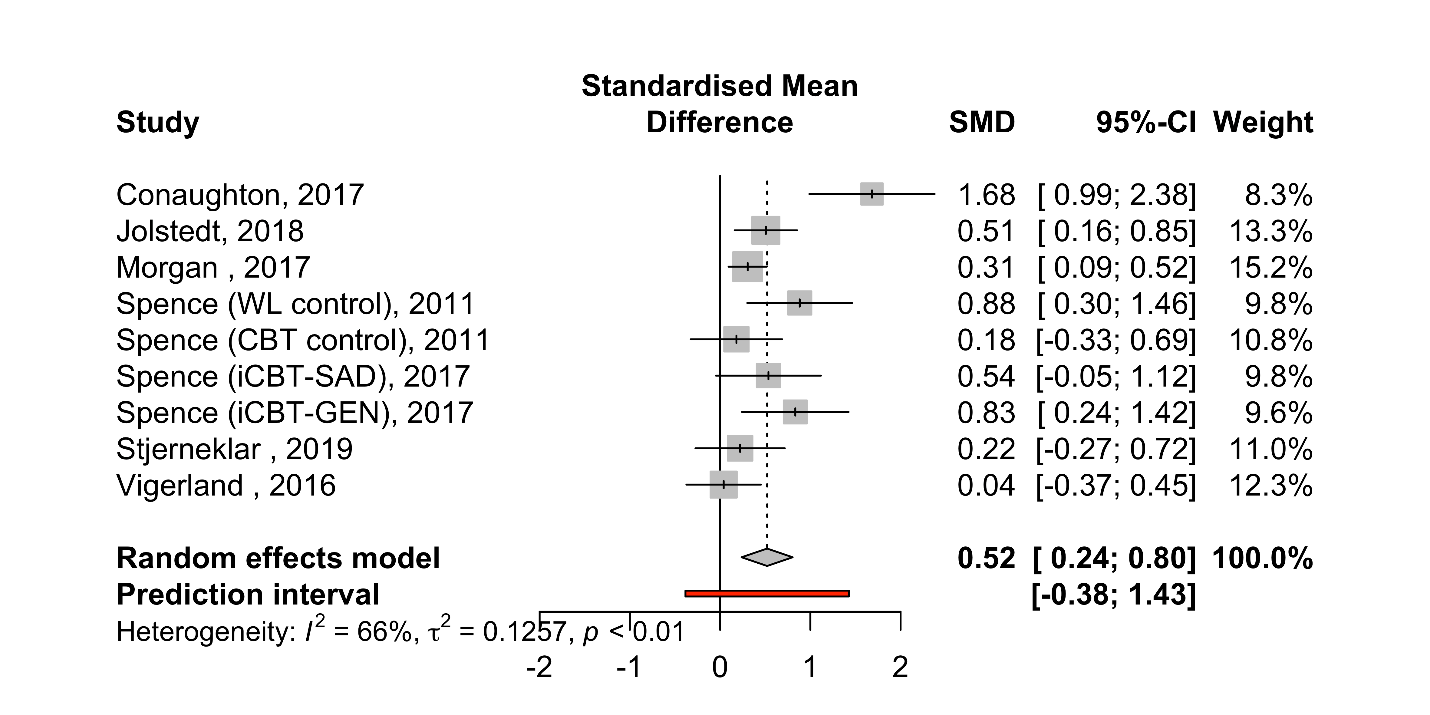


Figure S2. Post-treatment standardized mean difference (Hedge’s g) between internet-delivered treatment and control groups for quality of life outcomes. SMD: standardized mean difference.


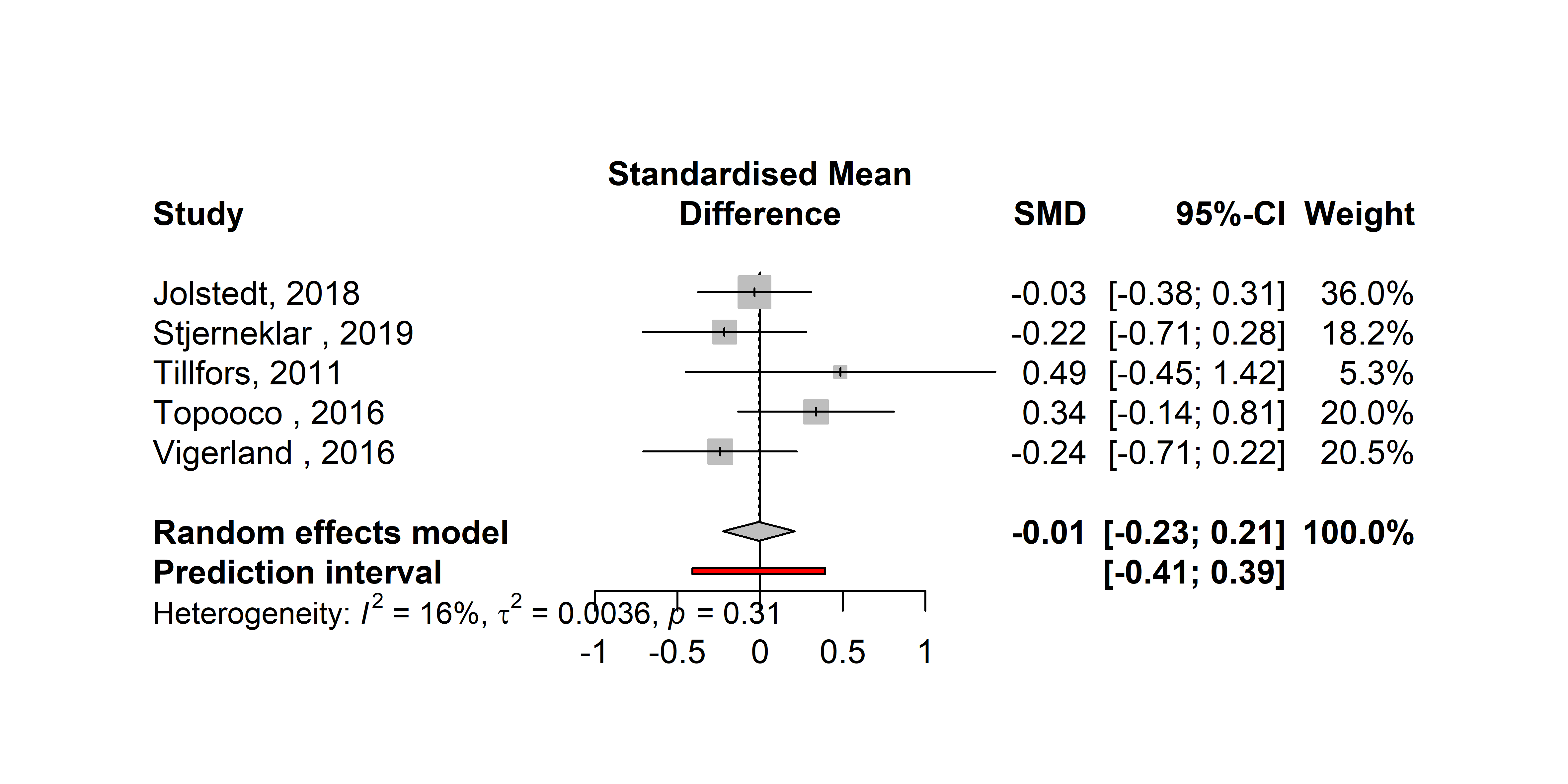


## Meta-analysis of follow-up outcomes

Figure S3. Follow-up standardized mean difference (Hedge’s g) between internet-delivered treatment and control groups for anxiety outcomes. GT: group treatment; NT: no treatment; PL: placebo; PWT-iCBM: picture-word training internet-delivered cognitive bias modification; SMD: standardized mean difference; ST-iCBM: scenario training internet-delivered cognitive bias modification; WL: wait-list.


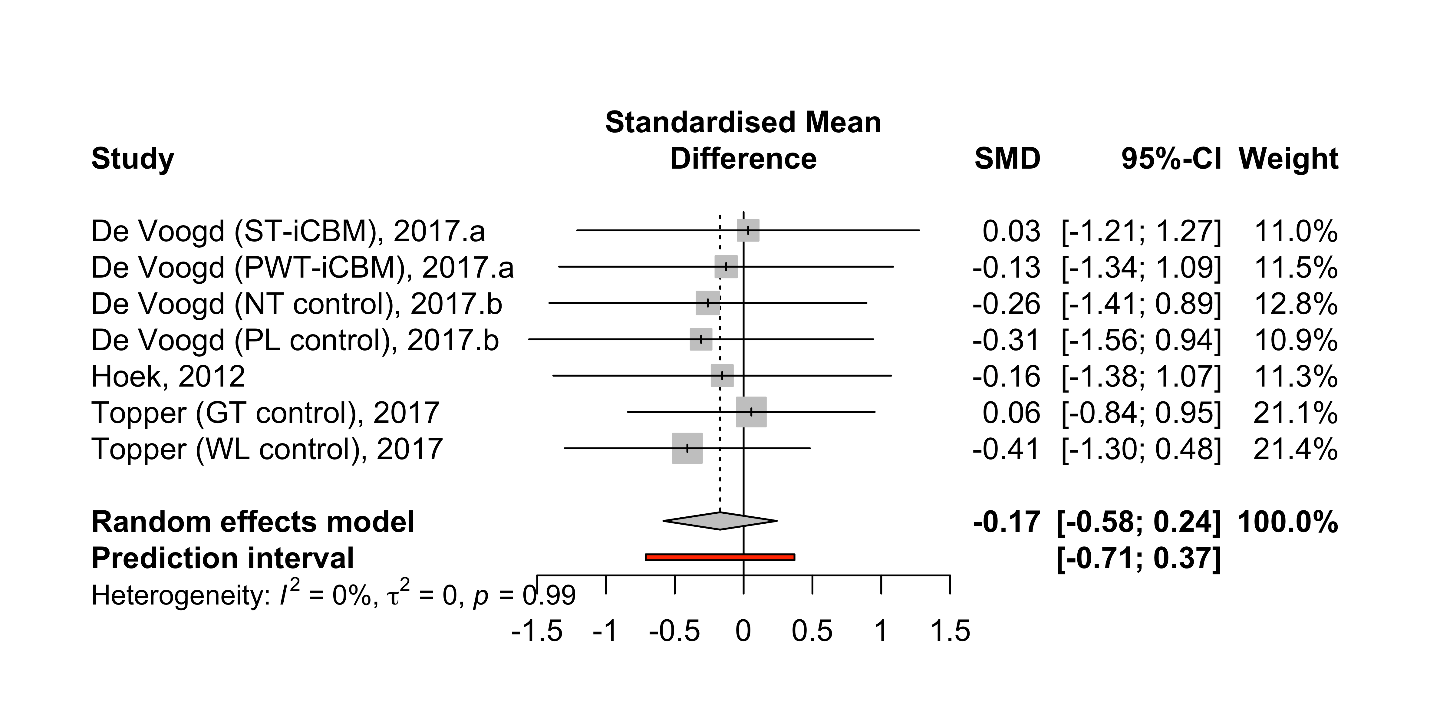


Figure S4. Follow-up standardized mean difference (Hedge’s g) between internet-delivered treatment and control groups for depression outcomes. GT: group treatment; NT: no treatment; PL: placebo; PWT-iCBM: picture-word training internet-delivered cognitive bias modification; SMD: standardized mean difference; ST-iCBM: scenario training internet-delivered cognitive bias modification; WL: wait-list.


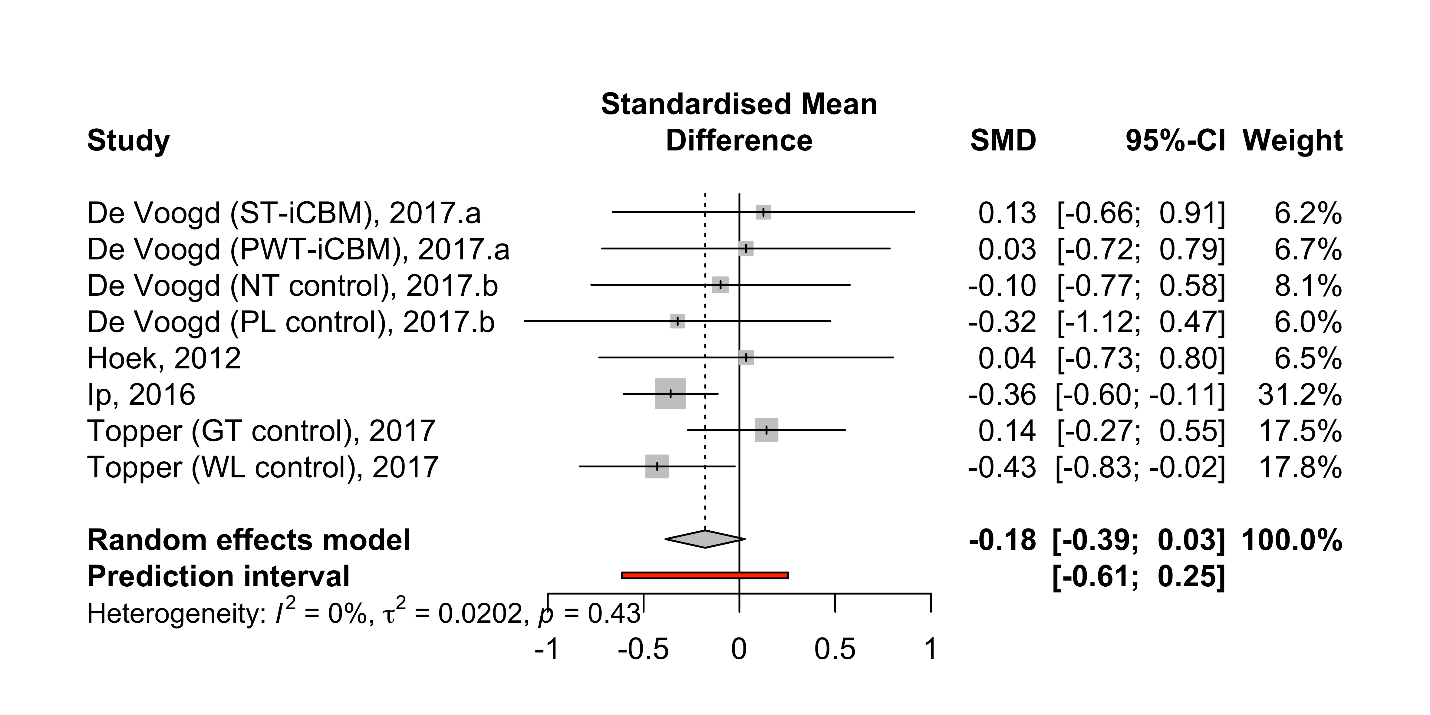


## Moderator analyses

Table S1. Mixed effect models.

| Moderator | Anxiety | Depression |
| --- | --- | --- |
|  | Q_(df)_, *P* | Q_(df)_, *P* |
| Effect size based on ITT vs on observed means | Q_(1)_=3.042, *P*=.081 | Insufficient data to conduct analyses |
| Passive vs active control group | Q_(1)_=0.535, *P*=.464 | Q_(1)_=1.553, *P*=.213 |
| Supported vs unsupported intervention | Q_(1)_=1.123, *P*=.288 | Q_(1)_=1.213, *P*=.271 |
| CBT-based vs non-CBT based intervention | Q_(1)_=0.244, *P*=.621 | Q_(1)_=0.086, *P*=.769 |
| Intervention delivered to  only youth vs youth and parent | Q_(1)_=0.005, *P*=.942 | All interventions were delivered to youth |
|  | Test of Moderators (coefficient 2):  F_(df1, df2)_, *P* | F_(df1, df2)_, *P* |
| Average age of sample | F_(1,18)_=0.082, *P*=.778 | F_(1, 11)_=1.64, *P*=.227 |
| Treatment length (in weeks) | F_(1,18)_=1.221, *P*=.283 | F_(1, 11)_=0.083, *P*=.778 |
| No modules | F_(1,18)_=0.811, *P*=.3797 | F_(1, 11)_=0.488, *P*=.499 |
| Percentage of sample female | F_(1,18)_=0.027, *P*=.872 | F_(1, 11)_=6.043, *P*=.032 |
| Average percentage of intervention completed at post-treatment | F_(1,18)_=1.149, *P*=.298 | F_(1, 11)_=0.064, *P*=.806 |

## Risk of Bias Assessment

Figure S5. Quality ratings for the risk of bias assessment of included studies.

##
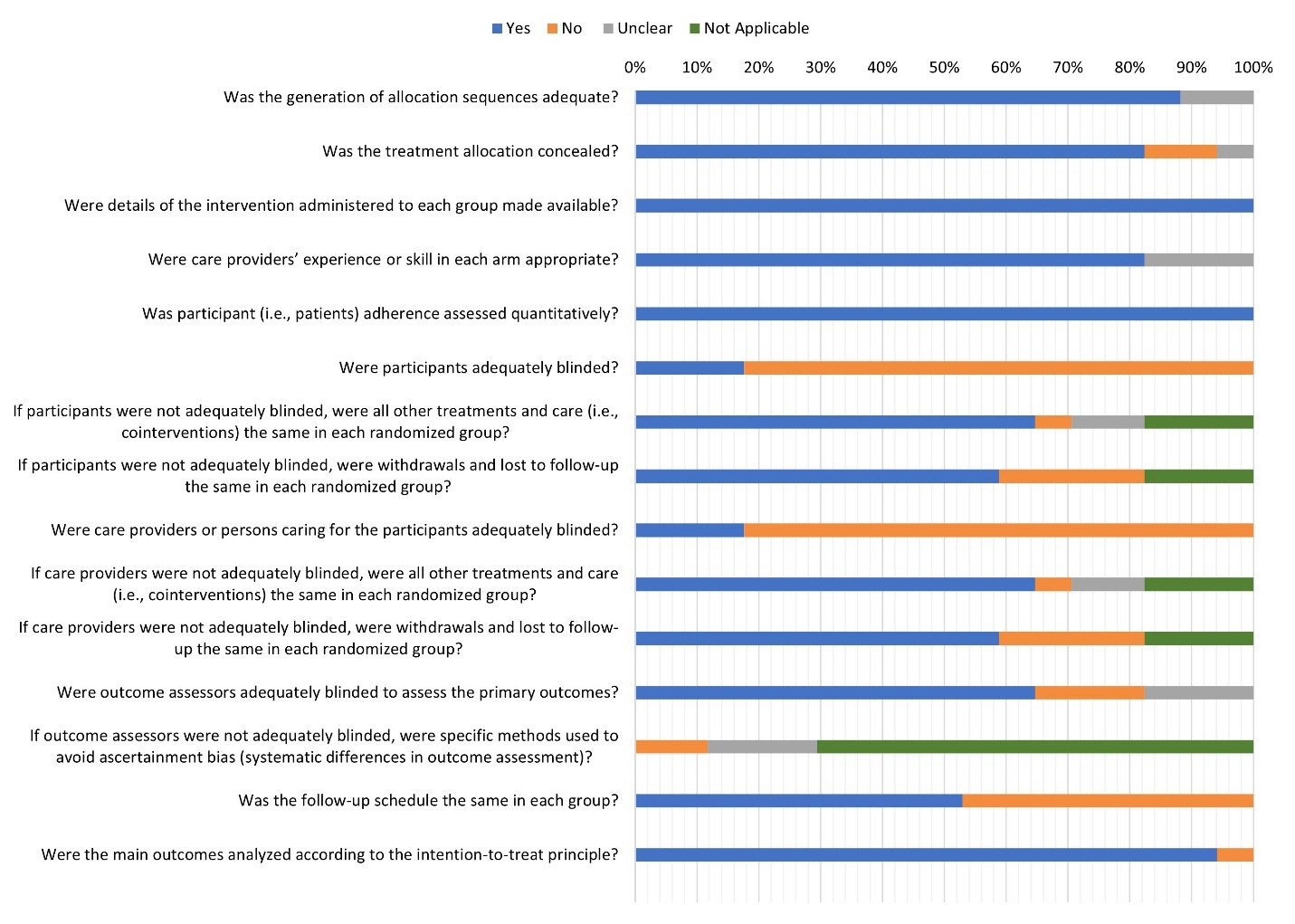


## Publication Bias Assessment

Figure S6. Funnel plot for anxiety (6a) and depression (6b) outcomes.


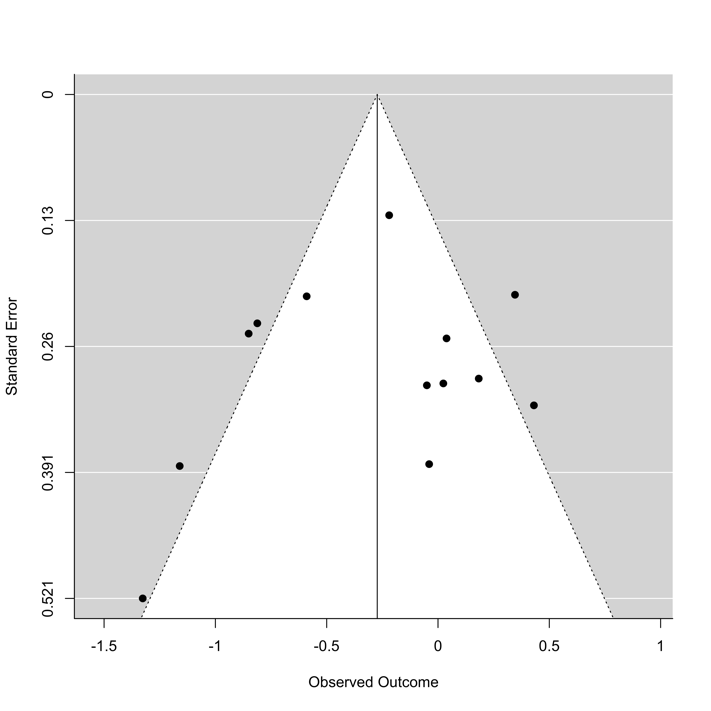

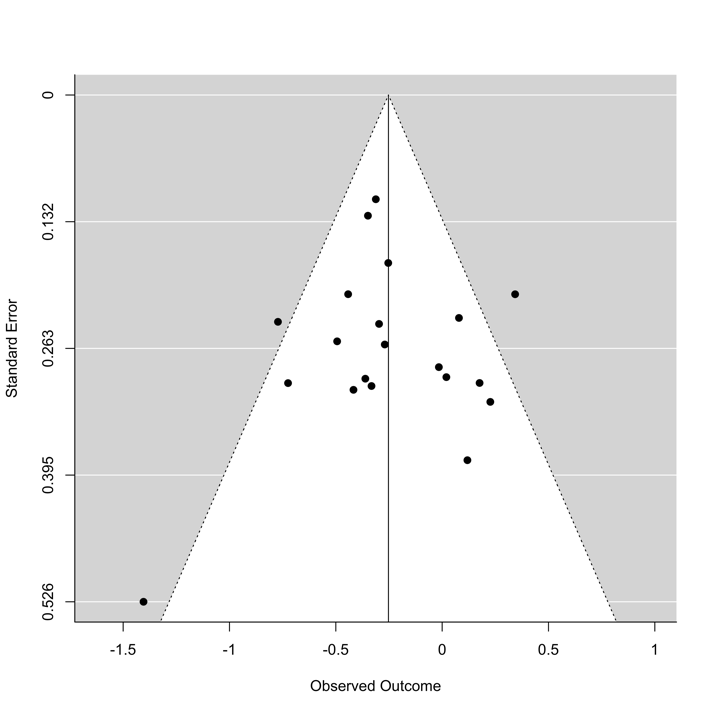


6b.a

6a.a
